# Endoscopic Closure Combined With the Endoscopic Submucosal Dissection Technique and Over‐the‐scope Clip for Chronic Aortoesophageal Fistula: A Case Report

**DOI:** 10.1002/deo2.70202

**Published:** 2025-09-02

**Authors:** Ryosuke Kawagoe, Toshiaki Narasaka, Takashi Mamiya, Masaaki Nishi, Kiichiro Tsuchiya

**Affiliations:** ^1^ Department of Gastroenterology Institute of Medicine University of Tsukuba Ibaraki Japan; ^2^ Graduate School of Comprehensive Human Sciences University of Tsukuba Ibaraki Japan; ^3^ Department of Gastroenterology Tsukuba Medical Center Hospital Ibaraki Japan

**Keywords:** aortoesophageal fistula, endoscopic closure, ESD, esophageal fistula, OTSC

## Abstract

Aortoesophageal fistula (AEF) is a rare but life‐threatening condition. Initial management typically includes thoracic endovascular aortic repair (TEVAR) or aortic graft replacement to achieve hemostasis, followed by esophagectomy with aortic graft replacement and greater omentum wrapping to eliminate the source of infection. We report a case of successful endoscopic closure of a chronic esophageal fistula secondary to AEF. A 63‐year‐old man presented with hematemesis. He had a history of two ascending aortic replacements and one descending aortic replacement for aortic dissection. Computed tomography revealed a pseudoaneurysm at the graft anastomosis site and perforation into the esophagus. He was diagnosed with AEF and underwent TEVAR for hemostasis. Although the bleeding was stopped, follow‐up imaging confirmed a residual fistula. Because of his poor general condition, surgery was contraindicated, and endoscopic closure was attempted. Initial treatment with hemostasis clips was ineffective. A second attempt using polyglycolic acid sheets, fibrin glue, and hemostasis clips with endoscopic submucosal dissection (ESD) achieved closure, but the fistula reopened after 5 months. Finally, the combination of ESD and over‐the‐scope clip (OTSC) achieved complete and sustained closure. The patient was discharged after 3 months but died 8 months postoperatively owing to idiopathic splenic rupture. This case demonstrates that the combination of ESD and OTSC may be an effective treatment option for chronic esophageal fistulas caused by AEF.

## 1 Introduction

Aortoesophageal fistula (AEF) is a rare but potentially fatal condition. The causes of AEF are classified into 2 categories: primary AEF, which arises from esophageal or aortic disease (eg, esophageal injury, esophageal cancer, aortic aneurysm, aortic dissection), and secondary AEF, which occurs as a complication of aortic surgery, such as thoracic endovascular aortic repair (TEVAR) or thoracic aortic graft replacement. The most common cause is postoperative status following aortic surgery, followed by aortic aneurysm, bone ingestion, and thoracic cancer. Initial treatment for secondary AEF is typically TEVAR or aortic graft replacement to achieve hemostasis, with TEVAR being preferred owing to its minimal invasiveness and certified hemostasis [1].

One of the major causes of death following successful initial treatment is persistent infection originating from the residual fistulous tract. To eliminate the infectious source, esophagectomy, open surgery with aortic replacement, and greater omentum wrapping are often performed to improve the prognosis [2]. Nevertheless, many patients with AEF are in poor general condition, making these surgical interventions infeasible in some cases.

Several reports have described endoscopic techniques for esophageal fistula closure, including hemostasis clip [3], over‐the‐scope clip (OTSC, Ovesco Endoscopy GmbH, Tübingen, Germany), fully covered self‐expandable metallic stent [4], polyglycolic acid (PGA) sheet (NEOVEIL SHEET, Gunze Medical, Osaka, Japan), fibrin glue filling [5], and the combination of endoscopic submucosal dissection (ESD) with hemostasis clip [6]. Currently, however, no consensus exists on the optimal closure method, and the treatment should be individualized according to the clinical context.

Herein, we report a case of secondary AEF following aortic surgery in which the patient was successfully treated with endoscopic fistula closure despite several challenges.

## 2 Case Report

A 63‐year‐old man presented to a previous hospital with hematemesis. He had a history of 2 ascending aortic replacements and 1 descending aortic replacement for aortic dissection and was taking warfarin. Computed tomography (CT) revealed a pseudoaneurysm at the graft anastomosis with perforation into the esophagus. Secondary AEF was diagnosed, and TEVAR was performed, resulting in successful hemostasis.

Esophagogastroduodenoscopy (EGD) was performed 14 days after the initial treatment and revealed a fistula approximately 5 mm in diameter in the middle thoracic esophagus. Esophagography revealed a periaortic cavity communicating with the esophageal fistula. The patient was started on an oral broad‐spectrum antibiotic (faropenem) for preventing graft infection due to intestinal bacterial translocation. Although there were no signs of active inflammation, he needed treatment for the fistula to prevent fatal infection. Radical treatment with esophagectomy and thoracic aortic graft replacement was considered; however, surgical intervention was deemed too high a risk given his history of multiple previous thoracic surgeries.

He was prohibited from oral intake for 1 month to promote spontaneous closure of the fistula, but it was unsuccessful. Although he underwent two endoscopic fistula closures with hemostasis clips, the fistula did not close. Three months after the initial treatment, he was referred to our hospital for endoscopic fistula closure.

On admission, his temperature was 36.8°C; blood pressure, 95/49 mm Hg; and heart rate, 68 bpm. The physical examination findings were unremarkable. The laboratory findings showed a white blood cell count of 6200/µL (normal), hemoglobin concentration of 11.3 g/dL (slightly decreased), C‐reactive protein of 0.62 mg/dL (slightly increased), and prothrombin time‐international normalized ratio (PT‐INR) of 1.69 (elevated). CT revealed no signs of active inflammation or abscess around the aorta.

**FIGURE 1:**
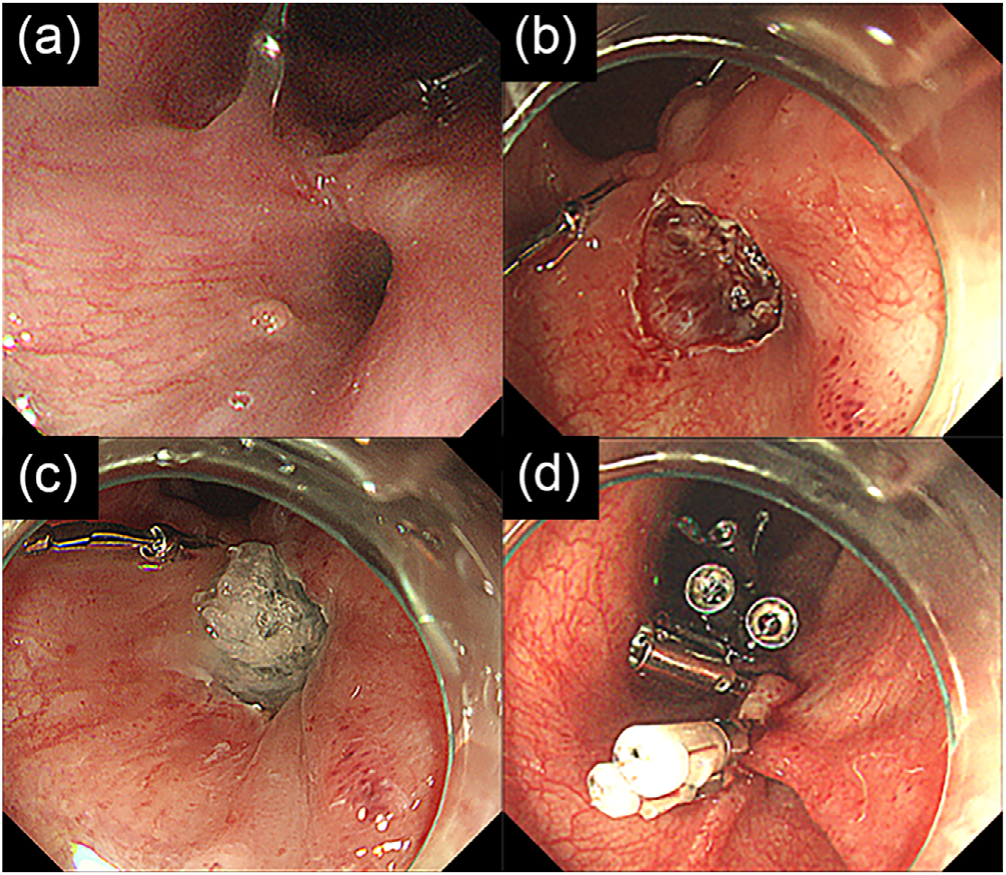
Images of the first endoscopic closure performed at our hospital. (a) A fistula was observed in the middle of the thoracic esophagus. (b) Submucosal dissection was performed around the fistula. (c) The ulcer was filled with polyglycolic acid sheets and fibrin glue. (d) The fistula was closed by use of hemostasis clips.

EGD showed epithelialization of the esophageal mucosa surrounding the fistula. We therefore performed submucosal dissection using the ESD techniques to create an artificial ulcer around the fistula, filled the ulcer with PGA sheets and fibrin glue, and closed the fistula using hemostasis clips (EZ Clip, Olympus, Tokyo, Japan, and SureClip, MC Medical, Tokyo, Japan) (Figure 1). EGD on postoperative day (POD) 6 and esophagography on POD 20 confirmed closure of the fistula, and he resumed oral intake. He was discharged 2 months postoperatively.

The follow‐up EGD at 5 months postoperatively, however, revealed fistula recurrence, and he was readmitted for suspected graft infection. After 2 months of fasting and antibiotic therapy, the infection resolved. Endoscopic closure using the same combination of ESD, PGA sheets, and fibrin glue filling, and hemostasis clips was attempted again, but the follow‐up EGD on POD 17 showed reopening of the fistula.

**FIGURE 2:**
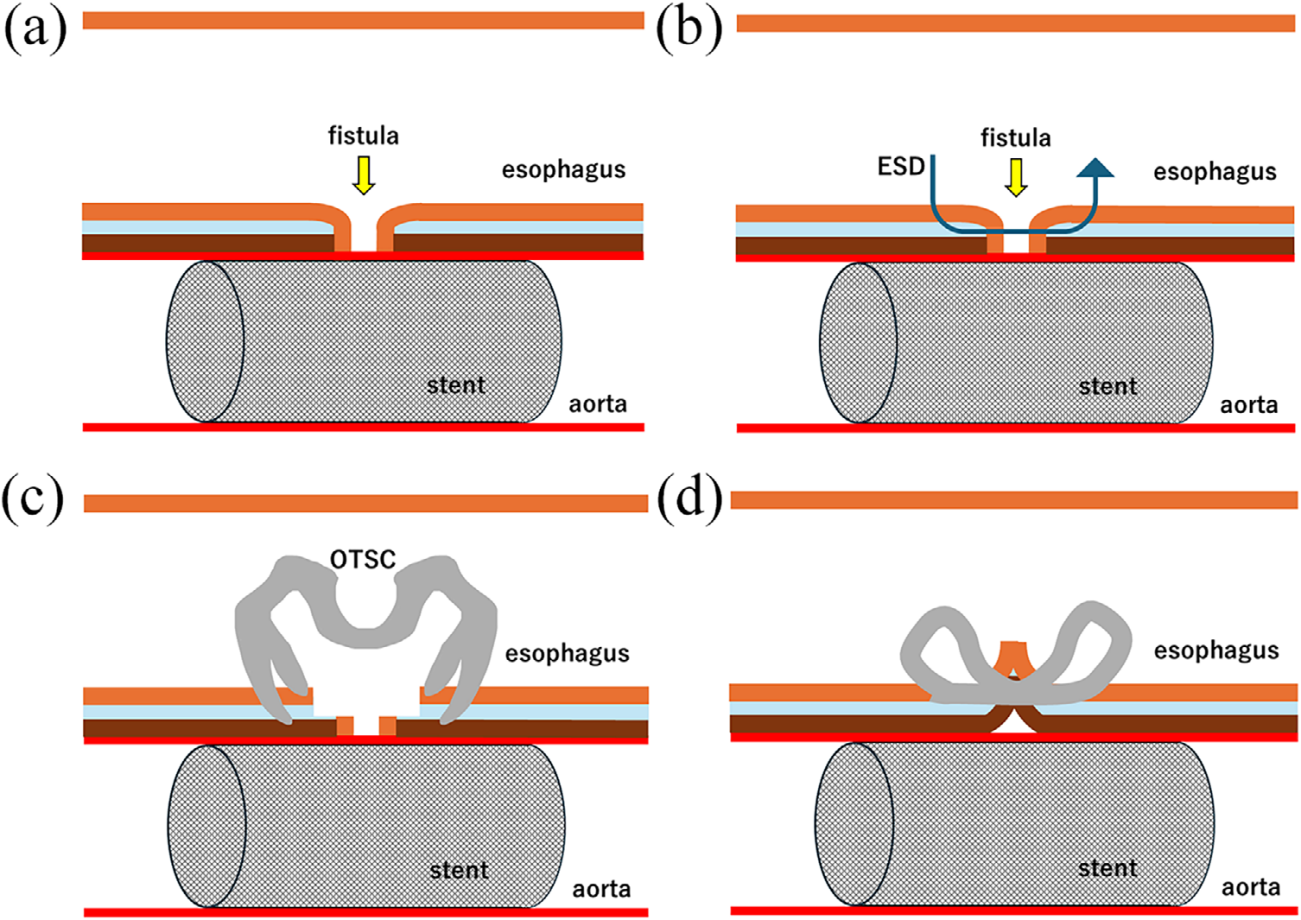
Schemes of chronic aortoesophageal fistula closure using the combination of endoscopic submucosal dissection and over‐the‐scope clip. (a) Schematic diagram of the aortoesophageal fistula. (b) Submucosal dissection of the esophageal mucosa around the fistula orifice. (c) Grasping the ulcer edge with an over‐the‐scope clip. (d) Complete closure of the fistula.

**FIGURE 3:**
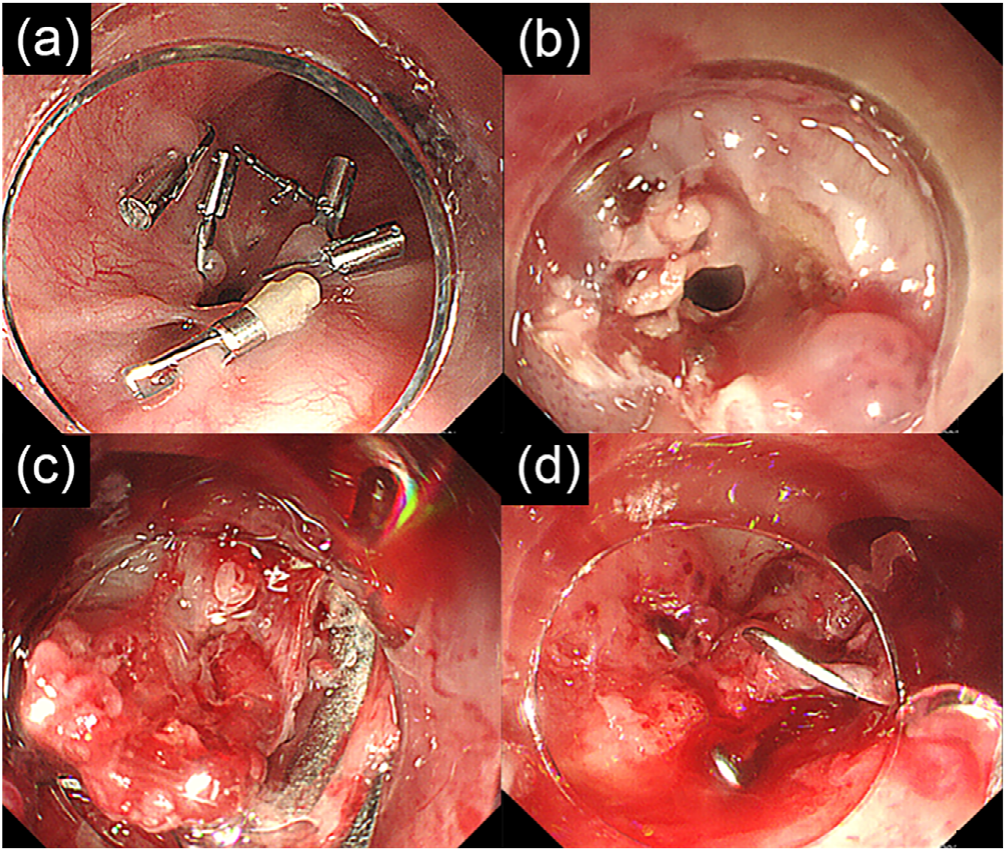
Images of the third treatment at our hospital. (a) The fistula was observed. (b) Submucosal dissection was performed around the fistula. (c) An over‐the‐scope clip was deployed. (d) Complete closure was achieved.

We therefore performed a third endoscopic treatment using the combination of ESD and OTSC placement (Figure 2). After injection of saline, the esophageal mucosa surrounding the fistula was incised using an apical knife (Dual knife J 1.5 mm, Olympus, Tokyo, Japan), followed by submucosal dissection. The edges of the fistula were grasped with a Twin Grasper (Ovesco Endoscopy GmbH, Tübingen, Germany), pulled into the hood, and a 10‐mm OTSC was deployed but slightly misaligned, resulting in incomplete fistula closure. An additional 9‐mm OTSC was placed at the partially open area, resulting in complete closure (Figure 3).

**FIGURE 4:**
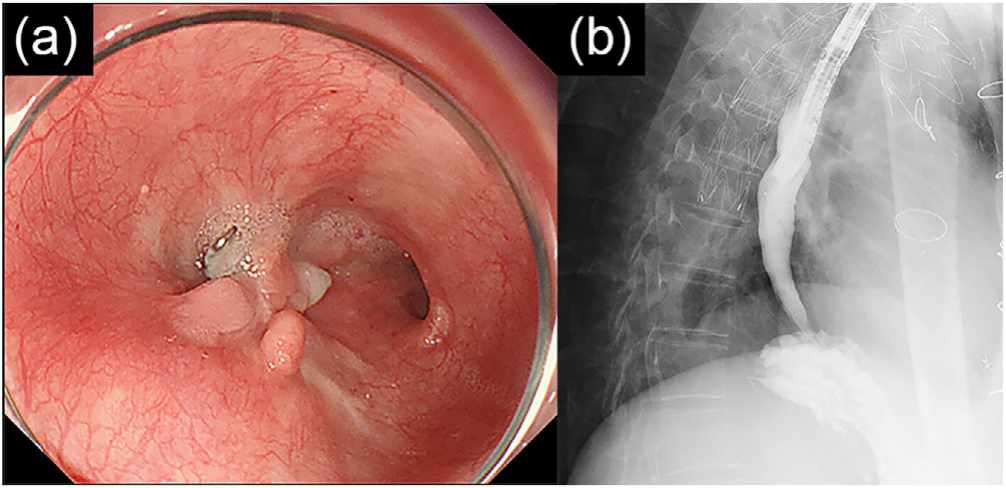
Images taken 6 months after the third treatment at our hospital. (a) The fistula remained closed. (b) Esophagography showed no periaortic cavity.

The follow‐up EGD on POD 4 confirmed closure of the fistula. Oral intake was resumed on POD 6, starting with water and followed by the gradual introduction of food. He was discharged 3 months postoperatively. The follow‐up EGD at 6 months postoperatively showed sustained closure, and esophagography confirmed no periaortic cavity (Figure 4); however, he developed idiopathic splenic rupture with associated renal failure and died 8 months after the final treatment.

## 3 Discussion

In acute fistulas occurring shortly after the onset of AEF, mucosal epithelialization has not yet developed. In such cases, closure may be achieved through natural tissue repair processes facilitated by hemostasis clips. However, the presence of massive bleeding and hemodynamic instability may make endoscopic closure difficult.

In contrast, chronic fistulas are characterized by epithelialization of the surrounding mucosa, making closure with hemostasis clips alone difficult. Creating an artificial ulcer may promote wound healing, and argon plasma coagulation (APC) [7] and ESD [8] have been reported to create such mucosal injury. However, ESD can perform more reliable mucosal ablation than APC and softens the edges of the fistula, facilitating clip closure and potentially improving closure outcomes.

PGA is a bioabsorbable reinforcement material that promotes granulation tissue formation. Several studies have reported the utility of combining PGA sheets with fibrin glue in the treatment of esophageal fistulas [9, 10]. A single‐center prospective study of patients with postesophagectomy esophageal fistula using PGA sheets reported successful closure in only two of five cases (40%), indicating limited efficacy [5]. To our knowledge, no reports have previously been published describing the combination of ESD, PGA sheet with fibrin glue filling, and hemostasis clips for the closure of esophageal fistulas. However, in this case, only a temporary closure was achieved, suggesting that a stronger closure force is needed for holding hard tissue such as chronic esophageal fistulas.

OTSC has a strong grasping force, allowing it to grasp hard tissue that is difficult with a conventional hemostasis clip. It is widely used for the management of gastrointestinal bleeding and fistula closure. However, in a case series on AEF closure with OTSC, all three cases had recurrence [4], suggesting OTSC alone may be insufficient. In this case, the combination of ESD and OTSC achieved sustained closure without recurrence for 6 months. No prior reports have described this combination for AEF. Creating an artificial ulcer with ESD may play a crucial role in improving closure outcomes. Although minimal fibrosis in this case, repeated interventions can make ESD challenging. OTSC was initially avoided due to stricture risk, but no such complication occurred. Thus, this method may be a first‐line endoscopic closure. Further cases are needed to validate its efficacy and safety.

In conclusion, the combination of ESD and OTSC was effective for closure of chronic esophageal fistula due to secondary AEF. This method may be a feasible treatment option for patients with chronic esophageal fistulas who are unsuitable for surgical intervention.

## Conflicts of Interest

Takashi Mamiya received honoraria from Viatris Pharmaceuticals Japan, AbbVie GK, Asahi Kasei Pharma Corporation, Mochida Pharmaceutical Co., Olympus Corporation, Nipro ES Pharma Co., Ltd., Bristol Myers Squibb, and Otsuka Pharmaceutical Co., Ltd., and received travel support for attending meetings from AbbVie GK. All other authors declare no conflicts of interest for this article.
